# Serum metabolomics strategy for understanding the therapeutic effects of Yin-Chen-Hao-Tang against Yanghuang syndrome†

**DOI:** 10.1039/c7ra11048k

**Published:** 2018-02-15

**Authors:** Xing-yuan Liu, Ai-hua Zhang, Heng Fang, Meng-xi Li, Qi Song, Jing Su, Meng-die Yu, Le Yang, Xi-jun Wang

**Affiliations:** Sino-America Chinmedomics Technology Collaboration Center, National TCM Key Laboratory of Serum Pharmacochemistry, Chinmedomics Research Center of State Administration of TCM, Laboratory of Metabolomics, Department of Pharmaceutical Analysis, Heilongjiang University of Chinese Medicine Heping Road 24 Harbin 150040 China; State Key Laboratory of Quality Research in Chinese Medicine, Macau University of Science and Technology Avenida Wai Long, Taipa Macau China xijunwangls@126.com

## Abstract

Yin-Chen-Hao-Tang (YCHT), a classic Chinese herbal formula, is characterized by its strong therapeutic effects of liver regulation and relief of jaundice, especially Yanghuang syndrome (YHS). YHS is a type of jaundice with damp-heat pathogenesis, and it is considered a complicated Chinese medicine syndrome (CMS). The accurate mechanism for healing YHS has not yet been completely reported. The purpose of the current research is to investigate the expression of endogenous biomarkers in YHS mice and evaluate the clinical therapeutic effect of YCHT. Serum samples were analyzed using UPLC-Q/TOF-MS techniques in order to determine differential metabolites to elucidate the functional mechanism of YCHT on YHS through metabolite profiling combined with multivariate analysis. Simultaneously, the exact diversification of YHS mice was elucidated using blood biochemistry indexes and histopathological examination, and the results indicated that YHS is markedly improved by YCHT. Unsupervised principal component analysis (PCA) patterns were constructed to dissect the variances of metabolic profiling. Overall, 22 potential biomarkers were identified using a metabolomics approach based on an accurate MS/MS approach, clustering and distinguishing analysis. The present work demonstrates that the effectiveness of YCHT against YHS prompts distinct discrepancies in metabolic profiles by adjusting biomarkers and regulating metabolic disorders. A total of 15 metabolic pathways were involved in biological disturbance. This demonstrates that metabolomic techniques are powerful means to explore the pathogenesis of CMS and the therapeutic effects of traditional Chinese formulae.

## Abstract 2

The purpose of the current research is to investigate the expression of endogenous biomarkers in Yanghuang syndrome mice and evaluate the clinical therapeutic effect of Yin-Chen-Hao-Tang.

## Graphical abstract

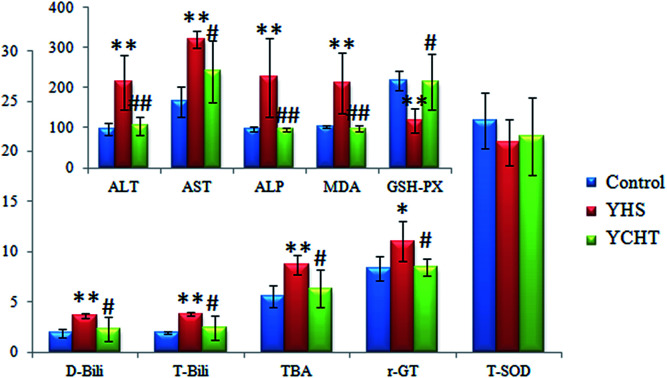

## 1. Introduction

Metabolomics is a novel and promising discipline in the post-genomics period, and it is a vital section of systems biology. It is used to investigate abnormal endogenous small molecule metabolites and their change regularity in biological systems.^1,2^ Metabolomics is defined as a promising omics method of exploring the overall alteration of biological systems, the research goal of which is to make qualitative and quantitative analysis of the dynamic changes of endogenous metabolites.^3^ Furthermore, the correlation between the changes of metabolites and the pathological and physiological processes of the organism can be determined. Nowadays, mass spectrometry plays an increasingly significant part in the study of metabolomics.^4,5^ Metabolomics has high requirements for sample processing, including the need to remove large molecular substances and recycle small molecular compounds as far as possible. Ultra-performance liquid chromatography combined with mass spectrometry (UPLC-MS) is a robust method for metabolic profile analysis of complex biological specimens in order to obtain potential biological information.^6^ The holistic system strategy of metabolomics coincides with the overall concept and ways of thinking of traditional Chinese medicine (TCM).^7,8^ It is widely applied in the field of TCM, and can possibly transform TCM from treatments based on experience into a modern medicine based on standards and scientific methods.^9–11^ Metabolomics provides a powerful analytical strategy for evaluation of the efficacy of TCM and study of the innate characteristics of complex Chinese medicine syndrome (CMS).^12,13^ The fully absorbing approach of metabolomics will accelerate the integration of TCM into contemporary life sciences, and promote the rapid development of TCM modernization accordingly. Due to the limits of global metabolite identification, a network-based approach combining independent metabolite features together is carried out to infer molecular pathways and components without identification.^14^ The Image Data Resource (IDR) combines various independent research metadata together for biological data query and analysis. The addition of metabolomics data into the IDR will generate a novel biotechnology system.^15^

Yin-Chen-Hao-Tang (YCHT), a famous herbal formula, consists of three medicinal herbs: *Artemisia capillaris* Thunb., *Gardenia jasminoides* Ellis and *Rheum officinale* Baill. It exerts the effects of clearing heat and eliminating dampness to aid liver regulation and jaundice remission, and it has a long history in China. Serum pharmacochemistry of TCM has been carried out to analyze the administered samples and to identify the effective components that have strong bioactivity in YCHT.^16^ YCHT has a comprehensive range of pharmacological effects, including anti-hepatic fibrosis, anti-inflammatory, hepatoprotective and cholagogic effects, and so on.^17^ Pharmacological research and clinical applications have shown that YCHT can be applied to cure chronic hepatitis B, liver cirrhosis, hepatic fibrosis and cholestasis liver injury.^18–20^ Recently, metabonomics and proteomics have been used to study the basic mechanisms of liver diseases.^21,22^ It was elucidated that YCHT exerts therapeutic effects *via* multiple pathways and multiple targets.^23^ At present, the research concerning the chemical composition and pharmacological activity of YCHT is more advanced,^24^ but disease status-based studies are rare, especially those on the functional mechanism. We have highlighted the therapeutic effects of YCHT on YHS mice, and investigated the molecular mechanism of the action of YCHT in the entire organism. The mechanism made use of the idea that the metabolic response of YCHT can be used as an evaluation criterion to specifically validate the YHS mice model based on all serum metabolites being quantified by the UPLC-Q/TOF-MS analytical platform. Therefore, our study will contribute to understanding how YCHT can ameliorate YHS.

Chinese medicine jaundice syndrome is divided into Yanghuang and Yinhuang syndromes. Yanghuang syndrome (YHS) was first chronicled in the classical monography of TCM called ‘Shang han lun’, and its clinical manifestations include fever, thirst and a bright yellow colour in the skin and sclera, which are special characteristics different from those of Yinhuang syndrome. YHS is classed as a complicated CMS. Damp-heat constitution, accompanied by a certain degree of the pathological features of liver injury and cholestasis, usually appears in YHS patients.^25^ A high resolution UPLC-MS technique was used to analyze the features of the metabolism level in YHS patients. Using a metabolomics method combined with multivariate statistics, 44 potential biomarkers were analyzed and determined in YHS patients’ urine.^26^ Nevertheless, the exact mechanism of YCHT against YHS has not been fully elucidated, which severely restricts the progress of research in the TCM field. The purpose of this current investigation was to explore the change regulation of potential biomarkers and evaluate the clinical therapeutic effects of YCHT.

## 2. Materials and methods

### 2.1. Chemicals and reagents

Acetonitrile of HPLC grade was supplied by Merck (Darmstadt, Germany). Formic acid (FA) of HPLC grade was provided by Kermel Chemical Reagent Company (China). Ultrapure water was supplied by Watsons. Olive oil was purchased from Zhongliang Food Marketing Co., Ltd (China). α-Naphthylisothiocyanate (ANIT) was procured from Jingchun Biochemical Technology Company (Shanghai, China). *Zingiber officinale* Rosc. was supplied by Harbin Tongrentang Drug Store (Harbin, China). Alcohol was supplied by Beijing Reagent Company (Beijing, China). The assay kits for alanine aminotransferase (ALT), aspartate aminotransferase (AST), alkaline phosphatase (ALP), total bilirubin (T-Bili) and direct bilirubin (D-Bili) were supplied by Zhongshengbeikong Biological Technology Co., Ltd (China), meanwhile, the assay kits for γ-glutamyl transpeptidase (γ-GT), glutathione peroxidase (GSH-Px), total superoxide dismutase (T-SOD), malondialdehyde (MDA) and total bile acid (TBA) were supplied by Jiancheng Biotechnology Institute (Nanjing, China). The herbal materials of YCHT were supplied by Harbin Tongrentang Drug Store (Harbin, China). Every medicinal material was validated by Prof. Xijun Wang, Department of Pharmacognosy of Heilongjiang University of Chinese Medicine. YCHT was prepared within our experimental studio as described in the preceding literature.^27^

### 2.2. Animal handling

Male Balb/c mice (weighing 20 ± 2 g) were purchased from Silaike Experimental Animal Co., Ltd. (Shanghai, China). The indoor temperature was regulated at 24 ± 2 °C and humidity was maintained at 70 ± 5%. The mice had free access to food and water. The mice were allowed to adapt to a new environment in metabolic cages for 7 days, with 12 hours of darkness and 12 hours of light each day, before administration. All of the animals were randomly divided into three groups, which were the control, YHS and YCHT groups, and every group had twelve mice. On the first day, the YHS and YCHT groups were orally administered *Zingiber officinale* Rosc. solution (0.013 g kg^−1^) in the morning and alcohol (12.5% (v/v)) in the afternoon, with quantities of 0.1 mL per 10 g bodyweight for fourteen days. Mice in the control group received aqua distillate at the same dose everyday. The YHS group was orally administered ANIT solution (0.1 mL per 10 g bodyweight, dissolved in olive oil) on the 15th (15 mg kg^−1^) and 16th (10 mg kg^−1^) days. From the 16th day, the YCHT group was orally administered YCHT solution (5 g kg^−1^, 0.1 mL per 10 g bodyweight) for seven days. The control group was orally administered aqua distillate under the same conditions everyday. The experimental procedures were approved by the Animal Care and Ethics Committee at Heilongjiang University of Chinese Medicine and all experiments were performed in accordance with the Declaration of Helsinki.

### 2.3. Sample collection and preparation

Blood was collected by pricking the eyeball and collecting the blood of the mice on the mornings of the 17th and 23rd days. Separation of the serum was performed by centrifuging at 3000 rpm min^−1^ for 15 min at 4 °C after standing for 30 min at 4 °C, and it was then stored at −80 °C until use. After blood collection, liver tissue was immediately collected and then put into 10% formalin for 24 h for histopathology analysis. The ALT, AST, ALP, T-Bili, D-Bili, γ-GT, GSH-Px, T-SOD, MDA and TBA kits were used to determine the concentrations of ALT, T-SOD, AST, ALP, T-Bili, GSH-Px, D-Bili, γ-GT, MDA and TBA in the blood. Quality control (QC) samples were collected from every mouse of each group, combined, and divided into several equal parts for use. The collection of QC samples was carried out at the same time as that of the blood samples. All procedures are completely in conformity with the developer’s guiding principle. The above experimental processes were conducted according to the Ethical Committee of Heilongjiang University of Chinese Medicine guidelines.

Serum samples were thawed prior to preparation at room temperature. 100 μL mouse serum was added to 400 μL methanol fixed in 1.5 mL micro tubes and stood for 30 min after vortexing for 10 s, then centrifuged at 13 000 rpm min^−1^ for 20 min at 4 °C for supernatant acquisition. 400 μL supernatant was dried using a vacuum drying method at 40 °C, and redissolved in 400 μL methanol (with ultrasound treatment for 30 minutes), and then centrifuged at 13 000 rpm min^−1^ for 20 min at 4 °C to obtain 100 μL supernatant. Finally, 4 μL serum samples were injected into UPLC-Q/TOF-MS equipment for metabolism detection. The preparation method for the QC samples is the same as the serum sample preparation method.

### 2.4. UPLC-Q/TOF-G2Si-HDMS experiments

#### 2.4.1. Chromatography

Chromatography was carried out on an ultra-high performance liquid chromatography (UPLC) system (Waters Corp., Milford, MA/USA) employing MassLynx™ software (V4.1 SCN901) for the comprehensive detection of serum samples. The separation was achieved using a completely new ACQUITY UPLC HSS T3 Column (100 mm × 2.1 i.d., 1.8 μm; Waters Corporation, Milford, USA). The column temperature stayed at 45 °C. The analysis used a gradient eluting method, where acetonitrile (A, 0.1% formic acid) and water (B, 0.1% formic acid) were adopted as the mobile phase, and the flow rate was maintained at 0.4 mL min^−1^. The sample injection volume was 4 μL. The gradient eluting conditions were as follows: 0–3 min, 1–10% A; 3–5 min, 10–20% A; 5–8.5 min, 20–40% A; 8.5–9.5 min, 40–99% A; 9.50–11.50 min, maintained at 99% A; 11.50–12.00 min, sharply declining from 99% to 1% A; maintained at 1% A for 3 min to balance the column. In order to verify and optimize the stability and repeatability of the UPLC-Q/TOF-MS system, we prepared QC samples from every mouse which included all of the serum data throughout the whole procedure.

#### 2.4.2. Mass spectrometry

The comprehensive analysis of samples was performed on a high-throughput G2Si high-definition mass spectrometer (Waters Q-TOF SYNAPT™, Waters Corp, Manchester, England). The parameter conditions in positive ionization scanning mode were as follows: the electrospray ionization (ESI) source was modulated with a capillary voltage of 3.0 kV and sample cone voltage of 30 V; the temperature of the source and desolvation were set at 110 °C and 350 °C, respectively; and the desolation gas flow rate was maintained at 800 L h^−1^ with a cone gas flow of 50 L h^−1^. In the negative ion mode: the ESI source was modulated with a capillary voltage of 2.5 kV and sample cone voltage of 30 V; the temperature of the source and desolvation were set at 110 °C and 350 °C, respectively; and the desolation gas flow rate was maintained at 800 L h^−1^ with a cone gas flow of 50 L h^−1^. All data were detected in centroid mode, and the mass scan range was from 50 to 1200 Da. In order to ensure precise and stable mass acquirement, we employed leucine enkephalin (1 ng μL^−1^, flow rate of 5 μL min^−1^) as a lock-mass solution to conduct online quality correction ([M + H]^+^ = 556.2771, [M − H]^−^ = 554.2615).

### 2.5. Metabolic profiling and metabolite analysis

All original data were fed into the Progenesis QI software for data dimension reduction and matrix acquisition. This software can automatically complete peak recognition, noise reduction, peak alignment, peak picking and other preprocessors, and finally the output matrix included the retention time, *m*/*z* value, and normalized peak area. In the ‘review compounds’ step of QI, the preprocessed data were imported into EZinfo software in the form of full components, which could also be filtered to select the identified components and the unidentified components. Therefore, pattern recognition analysis such as the unsupervised PCA was carried out. All massive data sets can be analyzed without discrimination by the PCA multivariate statistical method, and the differences between groups seem transparent.

### 2.6. Biomarker identification and metabolic pathway analysis

Molecular ions were acquired from the UPLC-Q/TOF-MS system, and then the precise molecular mass was obtained within the range of deviation. HMDB and KEGG retrieval databases, combined with MS/MS information, were used to assess potential compounds according to their molecular weight and molecular formula, and finally determine the chemical structures of biomarkers *via* chromatographic retention behavior and MS/MS data. The serum metabolism profiles of mice were analyzed using the chinmedomics method, and potential biomarkers were found subsequently by analyzing metabolites that altered the metabolic profile. MetPA is a powerful tool based on networks and easy manipulation to analyze the metabolic pathways in organisms.^28^ MetPA combines several advanced path analysis procedures such as KEGG to analyze the topological characteristics of metabolic pathways, which can be more accurate in determining the metabolic pathways with the strongest correlation with analytical metabolites. The analysis results are presented in a network system similar to Google maps. When you click on any metabolite node, you can get the name, report, and various univariate statistical analyses of the relevant metabolites. Potential biomarkers were imported into MetPA to find the metabolic pathways closely associated with YHS. MetPA also offers clustering and visualization means to establish heatmaps, dendrograms and so on.

### 2.7. Statistical analysis

All of the metabolic data of this experiment were processed using SPSS software (version 18.0 for Windows, IBM, Chicago, IL), and were represented as mean ± SD. Student’s *t*-test is an equation test based on a single variable. The *P* value of the *t*-test was less than 0.05, which was considered statistically significant between the control and model group. Therefore, the serum data of the control group and the YHS group were processed using TTEST, the *P* value of which was less than 0.05 for a potential biomarker.

## 3. Results

### 3.1. Biochemical analysis

**Fig. 1:**
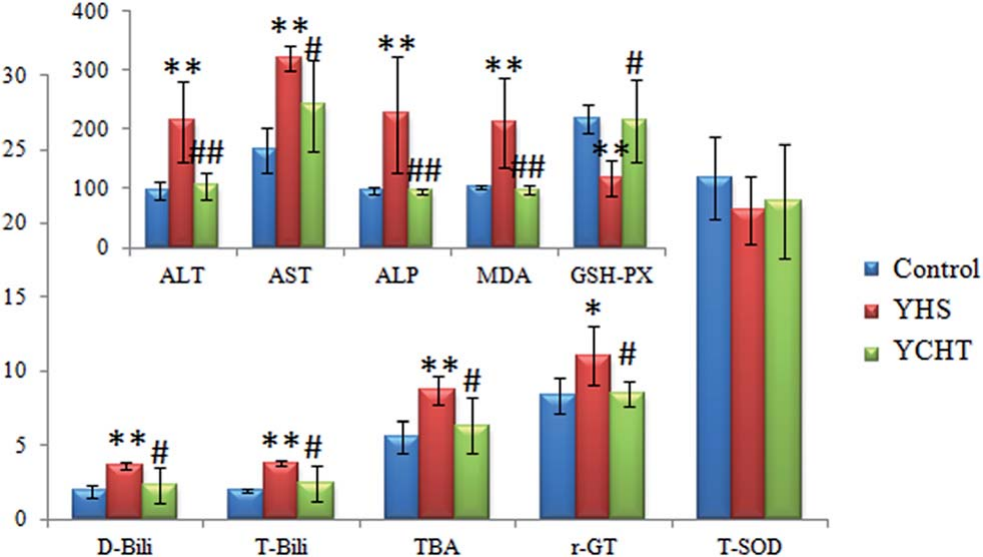
Biochemical analysis of the therapeutic effects of YCHT against Yanghuang syndrome. All of the results are expressed as mean ± SD. (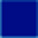) Control, (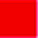) YHS and (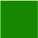) YCHT. The YHS group compared with the control group: *p < 0.05, **p < 0.01; and the YCHT group compared with the YHS group: #*p* < 0.05, ##*p* < 0.01. The specific values and units are shown in ESI Table 1.†

AST, ALT and ALP are mainly distributed in hepatocytes, and when the hepatocytes are damaged or undergo necrosis, all of the values of those will evidently increase. The amounts of ALT, AST, ALP, T-Bili, D-Bili and TBA in the serum of the YHS group were more significantly increased (*P* < 0.01) compared to those of the control group, the content of γ-GT was observably increased (*P* < 0.05), the content of T-SOD was decreased, the content of MDA in liver tissue was significantly increased (*P* < 0.01) and the content of GSH-Px was remarkably decreased (*P* < 0.01) (ESI Table 1†). The palpable content changes between the control and the YHS mice indicated that biological disturbance may have occurred in the YHS group. The amounts of ALT and ALP in the YCHT group were markedly decreased (*P* < 0.01) compared to those of the YHS group, the amounts of AST, T-Bili, D-Bili, TBA and γ-GT were evidently decreased (*P* < 0.05), the content of T-SOD was increased, the content of MDA in liver tissue was notably decreased (*P* < 0.01) and the content of GSH-Px was prominently increased (*P* < 0.05) (Fig. 1). These specific comparisons certified that YCHT treatment leads to a prominent improvement in YHS mice.

### 3.2. Histopathological results

**Fig. 2:**
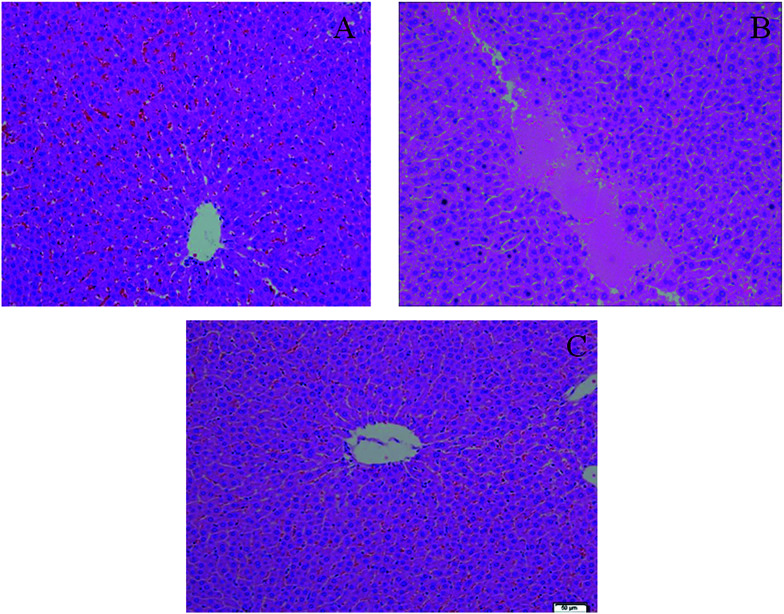
H & E staining in the therapeutic study of YCHT against Yanghuang syndrome. Control group (A), YHS group (B) and YCHT group (C) (magnification ×100). The structure of the hepatic lobule in the control group is complete, the hepatocytes are arranged radially in a central vein, there is dyeing uniformity, and the shape of hepatocytes is normal. But hepatocytes in the YHS group showed significant changes according to the histopathological observation. In particular, a large area of focal necrosis, ballooning degeneration and inflammatory cell infiltration are detected, and the liver tissue shows homogeneous powder staining in the YHS group compared with the control group. The shape of the hepatocytes is normal and the outline of the sinus hepaticus is clear in YCHT group mice compared with the YHS group.

YHS patients usually develop metabolic disorders in the liver as a direct result of hepatic diseases, which can induce serious histopathological changes. Histopathological results indicated that the structure of the hepatic lobule in the control group was complete, the hepatocytes were arranged radially in a central vein, there was dyeing uniformity, and the shape was normal (Fig. 2). But that of the YHS group showed significant diversity according to the histopathological detection. In particular, a large area of focal necrosis, ballooning degeneration and inflammatory cell infiltration were observed, and the liver tissue showed homogeneous powder staining in the YHS group compared with the control group. This demonstrated that YHS mice had sustained cholestatic liver injury. The shape of the hepatocytes was normal and the outline of the sinus hepaticus was clear in the YCHT group mice compared with the YHS group (Fig. 2).

### 3.3. Multivariate statistical analysis

**Fig. 3:**
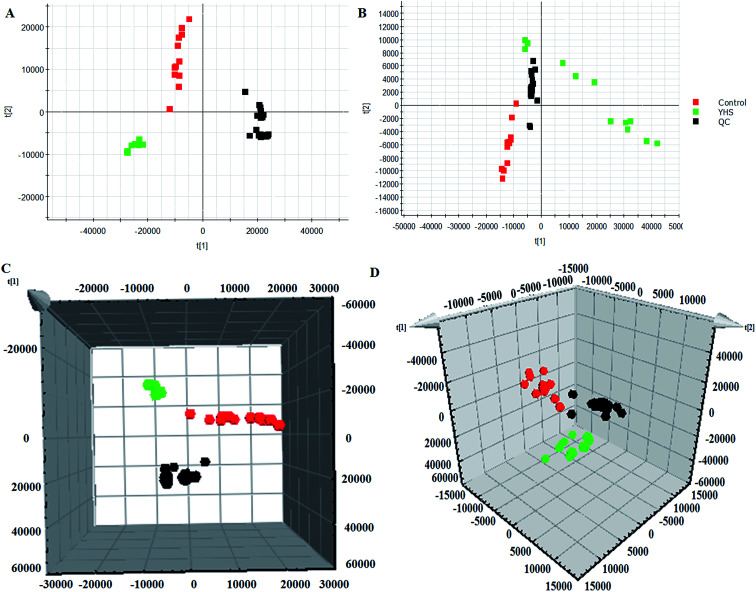
Multivariate data analysis of the serum metabolite data. 2D score plot of PCA showing the control group (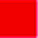), YHS group (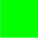) and QC samples (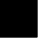) in the positive mode (A) and negative mode (B). 3D score plot of PCA in the positive mode (C) and negative mode (D).

Unsupervised PCA analysis was performed on the serum data of the control and YHS groups, for the sake of acquiring preliminary information about the metabolic profiles in the YHS group. From the 2D PCA score plots, the metabolic profiles of the YHS group separated evidently from those of the control group, in a direction away from the blood samples of the control group in both the positive and negative modes (Fig. 3A and B). The 3D PCA score plots suggested that the separation between the control and YHS groups was more distinct (Fig. 3C and D). The QC samples were included in the analysis in order to investigate the stability of the samples and the instrument. This indicated that a huge biological disturbance noticeably occurred in the metabolic process of the YHS group according to the metabolic alterations among the two groups.

### 3.4. Identification of biomarkers

**Fig. 4:**
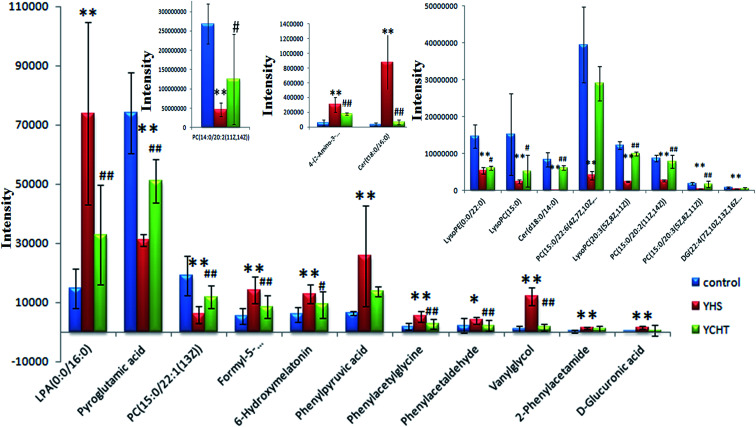
Relative signal intensities of serum metabolic biomarkers identified using UPLC/MS. Data are expressed as mean ± SD. The YHS group compared with the control group: *p < 0.05, **p < 0.01; and the YCHT group compared with the YHS group: #*p* < 0.05, ##*p* < 0.01.

In order to discover endogenous biomarkers that are essential in cluster classification and to reveal the characteristic changes of metabolic profiles effectively, we employed a UPLC-Q/TOF-MS technique combined with a pattern recognition analysis method to determine the serum biomarkers of YHS. In addition, the serum data of the control group and the YHS group were processed using a *t*-test, the *P* value of which was less than 0.05 for a potential biomarker. Finally, 22 metabolites were expressed as potential biomarkers that indicate the separation between the control and YHS groups (Fig. 4, ESI Table 2†). Among these, 13 metabolites were remarkably regulated by YCHT treatment. The relative signal intensities and the variance for each potential biomarker are listed in ESI Table 3.†

### 3.5. Metabolic pathway analysis

**Fig. 5:**
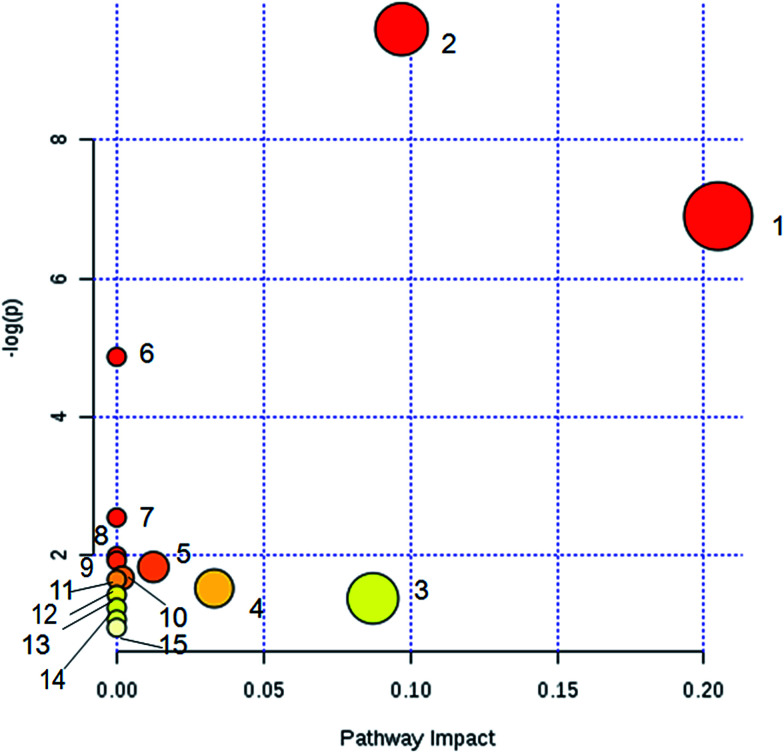
Metabolic pathway analysis of serum biomarkers. (1) Glycerophospholipid metabolism; (2) phenylalanine metabolism; (3) pentose and glucuronate interconversions; (4) ascorbic acid metabolism; (5) glycerolipid metabolism; (6) tryptophan metabolism; (7) linoleic acid metabolism, (8) biosynthesis of phenylalanine, tyrosine and tryptophan; (9) alpha-linolenic acid metabolism; (10) glutathione metabolism; (11) phosphoinositol metabolism; (12) starch and sucrose metabolism; (13) arachidonic acid metabolism; (14) tyrosine metabolism; (15) amino sugar and nucleotide glucose metabolism.

The identified biomarkers of YHS were imported into the MetPA website to analyze the relevant metabolic pathways. A total of 15 metabolic pathways were involved in the disturbance, including glutathione metabolism, glycerolipid metabolism, pentose and glucuronate interconversions, glycerophospholipid metabolism, phenylalanine metabolism, tyrosine metabolism, starch and sucrose metabolism, arachidonic acid metabolism, ascorbic acid metabolism, tryptophan metabolism, linoleic acid metabolism, biosynthesis of phenylalanine, tyrosine and tryptophan, phosphoinositol metabolism, alpha-linolenic acid metabolism, and amino sugar and nucleotide glucose metabolism (Fig. 5). These results evidenced that endogenous metabolites cause a strong disturbance in the overall metabolic profiles that are closely connected with YHS. The precise relevant metabolic pathways were mainly glycerophospholipid metabolism, phenylalanine metabolism and pentose and glucuronate interconversions.

### 3.6. Therapeutic effects of YCHT against YHS

**Fig. 6:**
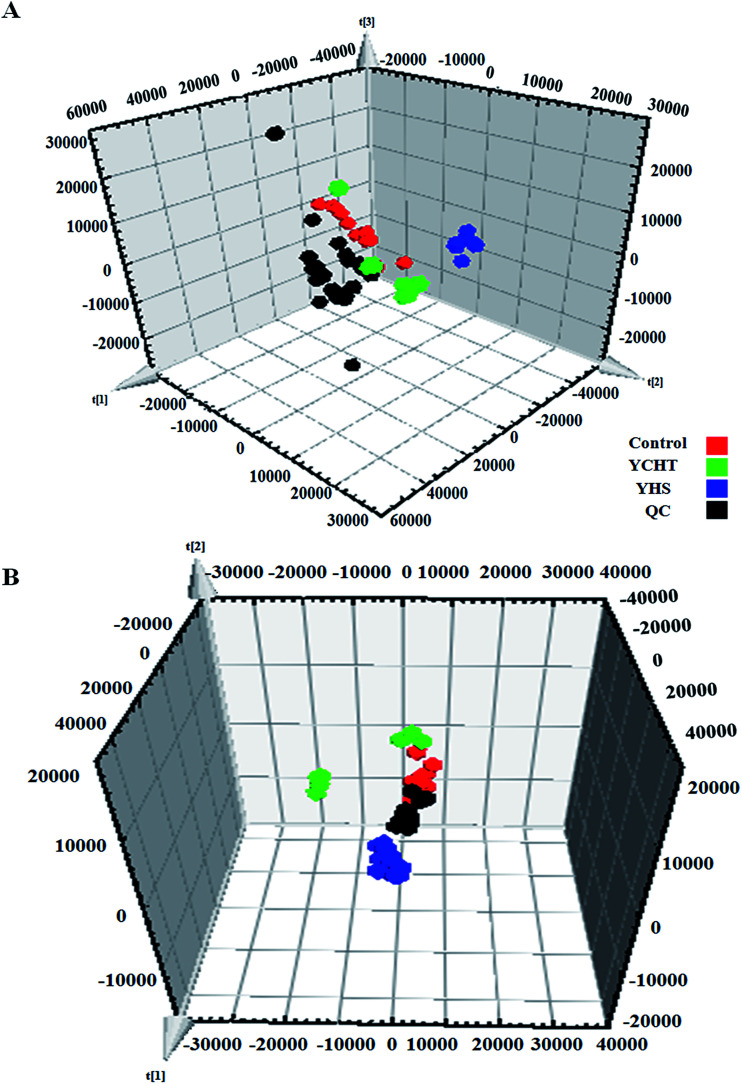
Pattern recognition analysis of the 3D score plot of PCA of YHS after YCHT treatment in the positive mode (A) and negative mode (B). (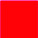) Control group, (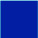) YHS group, (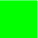) YCHT group, and (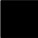) QC samples.

**Fig. 7:**
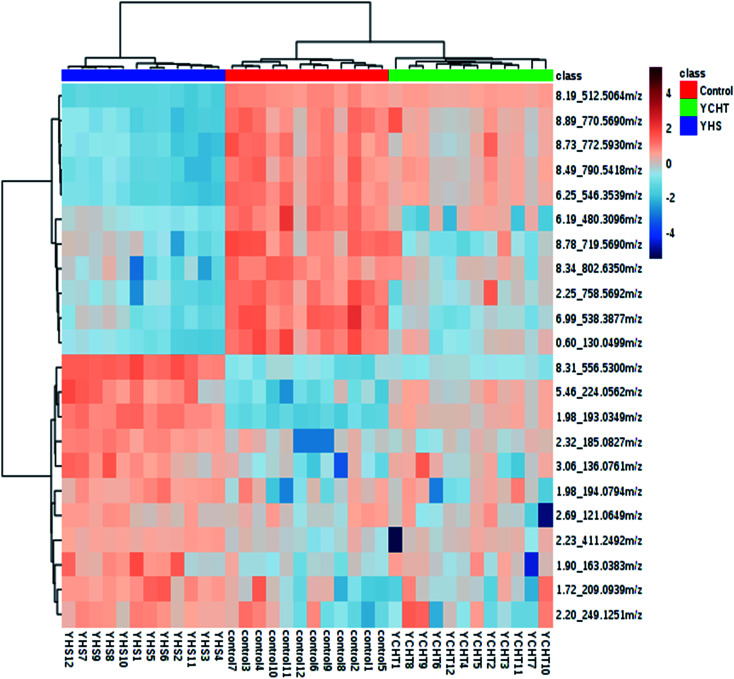
Heat map analysis of potential biomarkers among the control, YHS and YCHT groups. The degree of change is marked by different colors: red denotes upregulation and blue denotes downregulation. Each column represents an individual sample, and each row represents a biomarker.

After the YHS mice were orally treated with YCHT, the conditions of the mice were fundamentally restored to the healthy state. Focusing on the analysis of the PCA score plots, the YCHT group expressed a similar tendency to the control group which was much closer than the YHS group in the 3D plot of PCA (Fig. 6A and B). The trend of regulation is more obvious, which indicated that YCHT might reverse the physiological changes of YHS. As shown in Fig. 7, the clustering heat map analysis of the potential biomarkers revealed the difference in the relative values between the control and YHS mice. In addition, it can directly reflect the callback effect of YHCT, which further illustrates the therapeutic effect of YHCT on YHS. In order to judge how YCHT affects YHS-induced metabolic alterations, the specific intensities of potential biomarkers were subsequently compared (Fig. 4, ESI Table 3†). The above results demonstrated that YCHT could exert a robust therapeutic effect on YHS mice, which was shown by the recovery to the normal level in most cases. Exploring perturbed metabolites and metabolic pathways could reveal the dynamic process of disease development. The relevant metabolic pathways mainly involved glycerophospholipid metabolism, pentose and glucuronate interconversions, phenylalanine metabolism and so on. All presented metabolic pathways were extremely relevant to the pathogenesis mechanism of YHS. In the liver, glucuronate combines with potential lipophilic toxicants through glycosidic bonds to produce more hydrosoluble substances for kidney excretion, which can be used to evaluate the liver’s ability to remove toxic substances or drugs.^29,30^ Bilirubin plays a vital role in liver metabolism, and the skin and sclera of YHS patients show different degrees of xanthochromia due to the elevated bilirubin levels in the blood. A serum metabolics study demonstrated that d-glucuronate is involved in the metabolic pathway of pentose and glucuronate interconversions, which may fundamentally contribute to elucidating the mechanism of YHS.

## 4. Discussion

A mass spectrometry-based metabolomics approach can give valuable information about the functional regulation and mechanistic insights of natural products,^31–33^ especially in the application of Traditional Chinese Medicine.^34^ Hepatocytes in the YHS group showed significant changes according to the histopathological results. Of note, a large area of focal necrosis, ballooning degeneration and inflammatory cell infiltration was observed in the YHS group. This implied that the YHS mice had already developed the symptoms of cholestatic liver injury. Based on a UPLC-Q/TOF-MS analytical platform with incorporated pattern recognition means, phenotypic characterization of YHS was conducted. 22 key metabolites were identified as potential markers that were essential in metabolic pathway regulation.

Phenylalanine is one of the essential amino acids in the human body, and it is an aromatic amino acid. The liver is the main site of phenylalanine metabolism, under normal circumstances, in addition to the synthesis of various proteins of tissue cells from amino acids. Tyrosine is mainly produced through metabolism in the liver and other tissues, and it subsequently converts to certain hormones and neurotransmitters in the nervous system and adrenal medulla. When pathological changes happen in liver tissue, phenylalanine metabolism leads to disorders. The circulation directly enters into the systemic circulation, and the concentration of phenylalanine and ketone in the blood are elevated, the clinical manifestation of which is phenylketonuria. Phenylpyruvic acid is a direct metabolite of phenylalanine, and the content of phenylpyruvic acid is closely related to tyrosinemia. Patients with acute tyrosinemia accompanied by hepatomegaly, hepatocyte fatty infiltration or necrosis, or some jaundice symptoms, and chronic patients, may have liver fibrosis, cirrhosis, and even liver cancer. The present study found that the levels of phenylpyruvic acid in the blood of the model group were significantly increased, suggesting that intracellular tyrosine aminotransferase caused abnormal metabolism due to hepatocyte damage, which induced tyrosinemia, and eventually led to the occurrence of YHS. At the same time, due to the rise in concentration of phenylalanine and ketones in the blood, it can effectively reflect the partial damage of hepatocytes, the components of which will also be released into the blood, including AST, which is necessary for metabolic conversion between phenylalanine and phenylpyruvic acid. AST is one of the gold standard indicators for clinical reaction of liver functional metabolism. It can be concluded from the experimental results that a large amount of AST is released into the blood, indicating that the increase of phenylpyruvic acid detected in the blood of the model group mice can effectively reflect the damage of hepatocytes *in vivo*, and the liver function was destroyed, while tyrosinemia and AST metabolic abnormalities prompted jaundice syndrome to be extremely likely to occur.

**Fig. 8:**
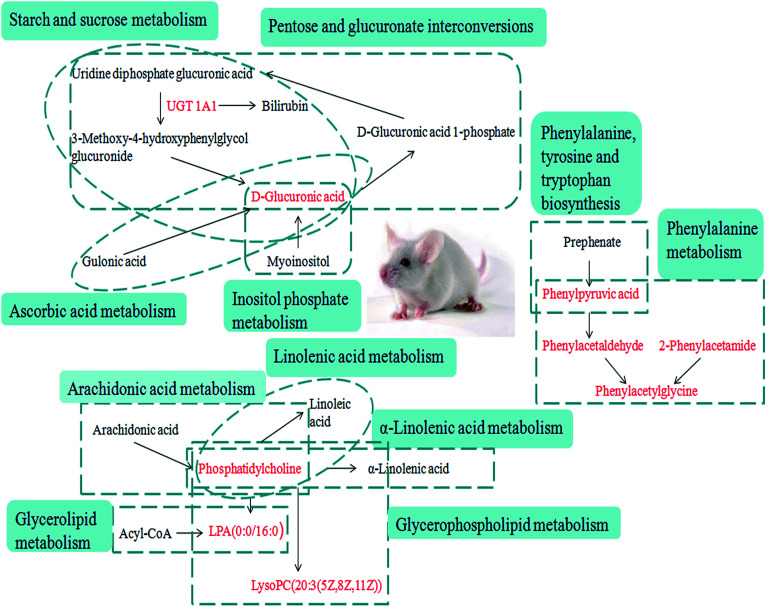
Correlation networks of all of the potential biomarkers on KEGG. The red font represents the biomarkers detected in this experiment.

The liver is a vital detoxifying organ in the human body. Toxic substances, which enter the blood through various channels in the body, are mostly metabolized by hepatocytes after entering the liver to produce non-toxic or low-toxicity compounds, which are then excluded from the body through kidneys or bile. The method of combination detoxification of glucuronate plays an important role in liver detoxification. Uridine diphosphate glucose produced by the process of glucose metabolism is further oxidized, and generates uridine diphosphate glucuronate (UDPGA), which is used as the supply of glucuronate. Under the catalytic conditions of UDP-glucurnosyl transferase 1A1 (UGT1A1) in a hepatic microsome, UDPGA binds the glucose in the molecule to the hydroxyl or amino group of toxic substances, thereby increasing its hydrosolubility, which is beneficial for the excretion of toxic substances. UGT1A1 is an important 2-phase metabolic enzyme in the organism, and it has similar tissue distribution to that of CYP450. It mainly exists in hepatic cytoplasm, and it can catalyze the glucuronidation of drugs, environmental toxicants, steroids and thyroid hormones, as well as promote the biosynthesis of glucoside in the brain. Meanwhile, it also participates in the biosynthesis of endogenous compounds such as bilirubin, bile acids, short-chain fatty acids and so on, and it is the key metabolic enzyme in the combination detoxification of glucuronate. The relevant networks of all of the potential biomarkers for YHS based on KEGG are expressed in Fig. 8.

Bilirubin has a certain degree of neurotoxicity, it can be cytotoxic at high concentrations, and it can cause irreversible damage to the brain and nervous system. Bilirubin is produced by erythrocytes. Erythrocyte senescence produces indirect bilirubin by the action of reductase. Indirect bilirubin is a small molecule lipophilic compound that enters the liver tissue with blood circulation to reversibly produce bilirubin-Y protein and bilirubin-Z protein by binding Y protein and Z protein in hepatocytes. It also links with UGT in a built-in network of hepatocytes by the action of glucuronate, where it is converted into direct bilirubin, and at this time direct bilirubin with high hydrosolubility is excreted *via* a series of reactions. d-Glucuronate is located in the metabolic pathway of pentose and glucuronate interconversion. Through focus analysis, we found that it is a downstream product of UGT1A1 metabolism, and subsequently we discovered that UGT1A1 is the only metabolic enzyme of bilirubin in the body. Indirect bilirubin must be converted into direct bilirubin through this enzyme, a process which allows the bilirubin to dissolve in the bile and be expelled from the body. The present study indicated that abnormal metabolism of d-glucuronate occurred in mouse serum from the YHS group, which may be directly related to the abnormal activity of UGT1A1. It was concluded that the abnormal metabolism of bilirubin was directly related to the increase of total bilirubin in the clinical biochemical indexes. After the treatment with YCHT, the levels of these evaluated indicators were significantly improved.

## 5. Conclusion

In our research, a robust chinmedomics strategy was established to explain the pathological changes of CMS and the therapeutic effect of TCM. Based on the most accurate collecting method, biological data were acquired and 22 potential biomarkers for YHS were detected under optimal conditions. These biomarkers are considered as possible drug targets, and 15 associated metabolic pathways were determined. Our study revealed that YCHT can reverse the pathological process of YHS by adjusting the perturbed metabolic pathway. Affirmatory biomarkers have underlying advantages for the clinical treatment of YHS, and this helps to boost the development of TCM modernization. In conclusion, using TCM theoretical instruction, a new model of YHS mice was established and it was evaluated using clinical biochemical indexing and histopathological diagnosis, clarifying the fundamentals of YHS from the perspective of metabonomics. Our research shows that serum metabonomics is a powerful strategy to thoroughly investigate CMS problems such as YHS, and it has great potential to clarify the essence of CMS.

## Conflicts of interest

The authors declare no competing financial interests.

## Supplementary Material

RA-008-C7RA11048K-s001
