# Supplementary material for: Serum metabolomics strategy for understanding the therapeutic effects of Yin-Chen-Hao-Tang against Yanghuang syndrome
Source: RSC Adv. 2018 Feb 15;8(14):7403–13. doi: 10.1039/c7ra11048k (PMC9078382; doi:10.1039/c7ra11048k)
Supplement: RA-008-C7RA11048K-s001 [file RA-008-C7RA11048K-s001.pdf]

Electronic Supplementary Material (ESI) for RSC Advances.
This journal is © The Royal Society of Chemistry 2018

Supplementary Table 1 The biochemical index analysis of control, YHS and YCHT groups

Group \ Index	ALT (U/L)	AST (U/L)	ALP (U/L)	D-Bili (μmol/L)	T-Bili (μmol/L)
Control	96.81 ±1.37×E	165.78 ±3.72×E	96.99 ±5.14	1.90 ±0.44	1.97 ±0.11
YHS	214.35 ±6.81×E**	320.89 ±2.13×E**	225.35 ±9.69×E**	3.62 ±0.25**	3.80 ±0.16**
YCHT	105.12 ±2.23×E##	240.50 ±7.64×E #	96.07 ±4.36##	2.29 ±1.18#	2.45 ±1.18#

Group \ Index	γ-GT (U/gprot)	TBA (μmol/L)	T-SOD (U/mgprot)	MDA (nmol/mgprot)	GSH-Px (μmol/L)
Control	8.34 ±1.17	5.58 ±1.09	23.07 ±2.79	102.49 ±4.01	217.29 ±2.33×E
YHS	11.02 ±1.96*	8.72 ±0.98**	20.93 ±2.31	212.37 ±7.50×E-**	117.63 ±2.95×E**
YCHT	8.46 ±0.80#	6.31 ±1.85#	21.51 ±3.81	97.98 ±7.22##	213.70 ±6.94×E#

The clinical biochemical index analysis results are expressed as mean±SD， n=12

*P<0.05, **P<0.01, control vs YHS; #P<0.05, ##P<0.01, YHS vs YCHT

Supplementary Table. 2 Identification and trends of serum potential biomarkers in YHS mice

NO	Rt (min)	m/z determined	m/z calculated	Error （mDa）	Ion form	Molecular Formula	Metabolite Name	Trend	T'TEST
1	8.73	772.5930	771.5778	0.2	$[M+H]^+$	$C_{43}H_{82}NO_8P$	PC(15:0/20:2(11Z,14Z))	↓	$1.594\times E^{-3}$
2	0.60	130.0499	129.0426	0.5	$[M+H]^+$	$C_5H_7NO_3$	Pyroglutamic acid	↓	$1.654\times E^{-2}$
3	2.20	249.1251	248.1161	2.9	$[M+H]^+$	$C_{13}H_{16}N_2O_3$	6-Hydroxymelatonin	↑	$4.540\times E^{-5}$
4	6.25	546.3539	545.3481	-2.8	$[M+H]^+$	$C_{28}H_{52}NO_7P$	LysoPC(20:3(5Z,8Z,11Z))	↓	$1.466\times E^{-6}$
5	8.19	512.5064	511.4964	3.1	$[M+H]^+$	$C_{32}H_{65}NO_3$	Cer(d18:0/14:0)	↓	$6.952\times E^{-9}$
6	8.78	719.5690	718.5536	-1.4	$[M+H]^+$	$C_{47}H_{74}O_5$	DG(22:4(7Z,10Z,13Z,16Z)/22:5(4Z,7Z,10Z ,13Z,16Z)/0:0)	↓	$1.981\times E^{-2}$
7	1.98	194.0794	193.0739	-0.1	$[M+H]^+$	$C_{10}H_{11}NO_3$	Phenylacetylglycine	↑	$4.578\times E^{-3}$
8	2.23	411.2492	410.2433	-3.4	$[M+H]^+$	$C_{19}H_{39}O_7P$	LPA(0:0/16:0)	↑	$4.933\times E^{-2}$
9	2.69	121.0649	120.0575	0.9	$[M+H]^+$	C_8H_8O	Phenylacetaldehyde	↑	$4.446\times E^{-3}$
10	3.06	136.0761	135.0684	2.7	$[M+H]^+$	C_8H_9NO	2-Phenylacetamide	↑	$2.989\times E^{-2}$
11	8.31	556.5300	555.5227	0.1	$[M+H]^+$	$C_{34}H_{69}NO_4$	Cer(t18:0/16:0)	↑	$5.923\times E^{-3}$
12	8.89	770.5690	769.5622	-0.5	$[M+H]^+$	$C_{43}H_{80}NO_8P$	PC(15:0/20:3(5Z,8Z,11Z))	↓	$1.618\times E^{-2}$
13	2.25	758.5692	757.5622	-0.3	$[M+H]^+$	$C_{42}H_{80}NO_8P$	PC(14:0/20:2(11Z,14Z))	↑	$3.516\times E^{-3}$
14	5.46	224.0562	223.0481	3.9	$[M+H]^+$	$C_{10}H_9NO_5$	4-(2-Amino-3-hydroxyphenyl)-2,4-dioxobutanoic acid	↑	$2.124\times E^{-7}$
15	6.99	538.3877	537.3794	1.9	$[M+H]^+$	$C_{27}H_{56}NO_7P$	LysoPE(0:0/22:0)	↓	$1.504\times E^{-2}$
16	1.72	209.0939	208.0848	-1.5	$[M+H]^+$	$C_{10}H_{12}N_2O_3$	Formyl-5-hydroxykynurenamine	↑	$3.473\times E^{-4}$
17	2.32	185.0827	184.0736	2.9	$[M+H]^+$	$C_9H_{12}O_4$	Vanylglycol	↑	$2.157\times E^{-3}$
18	8.34	802.6350	801.6248	4.6	$[M+H]^+$	$C_{45}H_{88}NO_8P$	PC(15:0/22:1(13Z))	↓	$1.049\times E^{-3}$
19	1.90	163.0383	164.0473	1.6	[M-H]-	$C_9H_8O_3$	Phenylpyruvic acid	↑	$3.070\times E^{-4}$
20	1.98	193.0349	194.0427	-2.6	[M-H]-	$C_6H_{10}O_7$	D-Glucuronic acid	↑	$4.211\times E^{-2}$
21	8.49	790.5418	791.5465	3.2	[M-H]-	$C_{45}H_{78}NO_8P$	PC(15:0/22:6(4Z,7Z,10Z,13Z,16Z,19Z))	↑	$4.768\times E^{-2}$
22	6.19	480.3096	481.3168	0.1	[M-H]-	$C_{23}H_{48}NO_7P$	LysoPC(15:0)	↓	$1.101\times E^{-2}$

↑Compared with control group, the level of metabolites in YHS group were increased
↓Compared with control group, the level of metabolites in YHS group were decreased

Supplementary Table. 3 Relative signal intensities of metabolic biomarkers.

NO	Groups / Biomarkers	Control	YHS	YCHT
1	PC(15:0/20:2(11Z,14Z))	8.66×E6 ±8.80×E5	2.66×E6 ±3.18×E5 **	7.83×E6 ±1.71×E6 ##
2	Pyroglutamic acid	7.40×E4 ±1.38×E4	3.11×E4 ±2.01×E3 **	5.10×E4 ±7.32×E3 ##
3	6-Hydroxymelatonin	5.99×E3 ±2.58×E3	1.28×E4 ±3.22×E3 **	9.36×E3 ±4.51×E3 #
4	LysoPC(20:3(5Z,8Z,11Z))	1.22×E7 ±1.07×E6	2.38×E6 ±2.37×E5 **	9.93×E6 ±5.14×E5 ##
5	Cer(d18:0/14:0)	8.39×E6 ±1.90×E6	3.85×E4 ±1.03×E4 **	6.11×E6 ±6.11×E5 ##
6	DG(22:4(7Z,10Z,13Z,16Z)/22:5 (4Z,7Z,10Z,13Z,16Z)/0:0)	8.64×E5 ±1.81×E5	3.72×E5 ±1.05×E5 **	4.64×E5 ±3.04×E5
7	Phenylacetylglycine	1.78×E3 ±1.17×E3	5.27×E3 ±1.97×E3 **	2.69×E3 ±1.68×E3 ##
8	LPA(0:0/16:0)	1.47×E4 ±6.80×E3	7.38×E4 ±3.07×E4 **	3.29×E4 ±1.67×E4 ##
9	Phenylacetaldehyde	2.20×E3 ±1.44×E3	3.99×E3 ±1.18×E3 *	2.22×E3 ±1.73×E3 ##
10	2-Phenylacetamide	4.03×E2 ±1.71×E2	1.51×E3 ±2.88×E2 **	1.03×E3 ±5.16×E2
11	Cer(t18:0/16:0)	3.39×E4 ±1.82×E4	8.80×E5 ±3.71×E5**	6.41×E4 ±2.99×E4 ##
12	PC(15:0/20:3(5Z,8Z,11Z))	1.85×E6 ±3.64×E5	3.74×E5 ±6.77×E4 **	1.73×E6 ±8.89×E5 ##
13	PC(14:0/20:2(11Z,14Z))	2.68×E8 ±5.19×E7	4.56×E7 ±1.74×E7 **	1.24×E8 ±1.16×E8 #
14	4-(2-Amino-3-hydroxyphenyl)-2, 4-dioxobutanoic acid	5.37×E4 ±4.06×E4	3.03×E5 ±1.02×E5 **	1.78×E5 ±1.48×E4 ##
15	LysoPE(0:0/22:0)	1.47×E7 ±3.14×E6	5.31×E6 ±8.74×E5 **	6.04×E6 ±5.43×E5 #
16	Formyl-5-hydroxykynurenamine	5.33×E3 ±2.68×E3	1.42×E4 ±4.48×E3 **	8.43×E3 ±3.82×E3 ##
17	Vanylglycol	9.96×E2 ±9.53×E2	1.19×E4 ±2.98×E3 **	1.69×E3 ±1.20×E3 ##
18	PC(15:0/22:1(13Z))	1.90×E4 ±6.71×E3	5.99×E3 ±2.83×E3 **	1.19×E4 ±3.92×E3 ##
19	Phenylpyruvic acid	6.49×E3 ±1.46×E3	2.57×E4 ±1.70×E4 **	1.36×E4 ±6.60×E3
20	D-Glucuronic acid	2.67×E ±4.62	1.58×E3 5.06×E2 **	5.04×E2 1.09×E2
21	PC(15:0/22:6(4Z,7Z,10Z, 13Z,16Z,19Z))	3.94×E7 ±1.02×E7	4.05×E6 ±1.07×E6 **	2.90×E7 ±4.48×E6
22	LysoPC(15:0)	1.52×E7 ±1.10×E7	2.41×E6 ±5.21×E5 **	5.25×E6 ±4.36×E6 #

Data are expressed as mean±SD. YHS group compared with control group: *p < 0.05, **p < 0.01; and YCHT group compared with YHS group: #p < 0.05, ##p < 0.01.
